# Eating Behaviors, Depressive Symptoms and Lifestyle in University Students in Poland

**DOI:** 10.3390/nu14051106

**Published:** 2022-03-06

**Authors:** Julia Suwalska, Kalina Kolasińska, Dorota Łojko, Paweł Bogdański

**Affiliations:** 1 Department of Treatment of Obesity, Metabolic Disorders and Clinical Dietetics, Poznan University of Medical Sciences, 60-569 Poznan, Poland; pbogdanski@ump.edu.pl; 2 Poznan University Hospital of Lord’s Transfiguration, 61-848 Poznan, Poland; kalina.kolasinska@skpp.edu.pl; 3 Department of Mental Health, Chair of Psychiatry, Poznan University of Medical Sciences, 60-572 Poznan, Poland; lojko@ump.edu.pl

**Keywords:** young adults, emotional eating, cognitive restraint, uncontrolled eating, depression, impulsivity

## Abstract

Young adulthood is the period from the late teens through the twenties and is associated with life transitions that could contribute to the development of obesity. Targeting this group will be critical to reversing the obesity epidemic. The aim of the study was to investigate the eating behaviors and lifestyle of healthcare students in Poland. We enrolled 227 students in the study. Convenience sampling was employed. Diet (Food Frequency Questionnaire), physical activity (International Physical Activity Questionnaire), depressive symptoms (Beck Depression Inventory), impulsivity (Barratt Impulsivity Scale), and eating behaviors (Three-Factor Eating Questionnaire) were assessed. One in three students exhibited depressive symptoms, one in four showed low levels of physical activity. More than 40% of the students did not consume vegetables at least once a day, and more than half did not consume fruit. Only approximately 50% of the students ate fish several times a month. There was an association between high scores of specific eating behaviors and body weight, adherence to the Mediterranean diet, and consumption of specific product groups (sweets, alcohol). The results of our study are expected to contribute to a better understanding of dietary habits and overweight/obesity in university students, and support the development of programs to promote healthy lifestyles in that population.

## 1. Introduction

Young adulthood, or emerging adulthood, is the period from the late teens through the twenties, with a focus on ages 18–25 [1]. This period is associated with many life transitions that could contribute to the development of obesity, including leaving home to live independently, studying, social networks, first job, marriage. Young adults transitioning from secondary education to university-level education experience profound changes in their lives, and their dietary habits deteriorate [2]. A low quality diet may be associated with micronutrient deficiencies. It was shown that young adults’ diets were below the recommendations for vitamins and minerals, particularly vitamin D, and overweight and obese participants had lower intakes of micronutrients than those of normal weight [3]. Furthermore, micronutrient deficiencies, including iron and vitamin B12, and apparent excess of folate were noted [4].

The risk of weight gain is greater during young adulthood than in any other period of life. In a study by Katsoulis et al., young adults had the most pronounced weight gain at 10 years (approximately 10 kg), with the highest absolute risk of transitioning from normal weight to excess weight or obesity [5]. The main cause of obesity and overweight is an energy imbalance between calories consumed and calories expended, resulting from an increased intake of energy-dense foods, high in fat and sugars, as well as physical inactivity [6]. However, it should be stressed that obesity is a complex and multisystem condition, resulting from the interplay of genetic predisposition, environmental context, and lifestyle choices and is, therefore, unlikely to be solved using simplistic causal models [7,8]. Many factors contribute to obesity at individual, interpersonal, environmental, and macrosystem levels [9]. Individual factors comprise, among others, impulsivity and eating behaviors, including emotional eating, uncontrolled eating, and cognitive restraint. Emotional eating (EE) is eating as a reaction to negative mood states and stress, serving as a temporary distraction from negative feelings [10,11,12]. This maladaptive coping strategy involves the consumption of high-calorie foods, thus, is associated with an increased risk of weight gain and obesity [13,14]. Uncontrolled eating (UE) is a tendency to overeat with the feeling of being out of control. UE may also occur in the absence of hunger [15]. Cognitive restraint or restrained eating is defined as the intention to restrict food intake to achieve or maintain a more desirable body weight [16]. Many studies show that restrained eaters/chronic dieters are more prone than non-dieters to binge eating. As physiological regulatory control is supplanted with cognitive control, the dieter is more vulnerable to disinhibition—anything that disrupts the dieter’s cognitive control or restraint unleashes overeating [17,18]. Impulsivity is a general tendency towards quick, unplanned reactions to internal or external stimuli, without considering the consequences of these actions to self or others [19]. Depression was found to be predictive of developing obesity, and obesity, to increase the risk of depression [20].

Studies on psychosocial, lifestyle, and genetic factors associated with obesity traits in undergraduate students have been carried out mainly in North America. In the review by Morassut et al. [21], European studies accounted for just 15%, with only two originating from Poland [22,23]. Similarly, Dietz et al. [24] found a strong research focus in the US, UK, and China, and a low number of original articles on modifiable health influencing factors in European countries, such as Portugal, Germany, Poland, and Italy.

A few interesting studies in young Poles have recently been published, including papers on eating behaviors among university students [25], eating habits and body composition of young Poles [26], and international studies on eating disorders, involving Polish samples [27,28]. However, the current body of evidence in the field of eating behaviors, diet, and physical activity among emerging adults in Poland and Central Europe is still scarce and more data are needed to develop preventive strategies.

Emerging adulthood is a key period for interventions, as it is a time of changes and transitions, during which students are potentially vulnerable to risky health behaviors, such as drinking or physical inactivity [24] and adverse changes in dietary patterns, eating behaviors, and food choices. On the other hand, healthy lifestyle habits formed during this period may have a sustaining impact on health across later life [29]. Our study aims at filling the gaps in knowledge, regarding Polish and Central European data. The objective of the study was to conduct a multidimensional assessment of weight-related factors in Polish healthcare students, considering the following: socioeconomic and demographic factors, diet, physical activity, eating behaviors, impulsivity, depressive symptoms. The results of our study are expected to contribute to a better understanding of dietary habits and overweight/obesity in university students, and support the development of programs to promote healthy lifestyles in that population.

## 2. Materials and Methods

### 2.1. Study Procedure and Participants

The study was designed as a cross-sectional study and conducted among undergraduate midwifery and physiotherapy students at the Poznan University of Medical Sciences. The sample size necessary to meet the statistical constraints was determined using the sample size calculator [30], which provided that the enrollment of 224 students would be needed. Convenience sampling was employed; students were approached at the end of one of their classes by a researcher and invited to the study. Out of 282 approached students, 235 agreed to participate (83.3% of invitees). All participants enrolled voluntarily and provided written informed consent before taking part in the study. The study was approved by the Ethical Committee of the Poznan University of Medical Sciences on 6 September 2018 by Resolution No. 857/18. All the procedures were carried out in compliance with the Helsinki Declaration. Data were collected between October 2019 and January 2020.

### 2.2. Applied Questionnaires

#### 2.2.1. Socio-Demographic Data

Self-reported socio-demographic and medical information was collected (age, sex, smoking, health status, etc.).

#### 2.2.2. Weight Status

Self-reported height and weight data were collected. The Body Mass Index (BMI) was calculated as the body mass in kilograms divided by the square of height in meters and categorized as underweight (BMI < 18.5 kg/m^2^), healthy weight (BMI 18.5 to 24.9 kg/m^2^), overweight (BMI 25.0 to 29.9 kg/m^2^), and obese (BMI 30.0 kg/m^2^ or higher) [31].

#### 2.2.3. Eating Behaviors

The 18-item Three-Factor Eating Questionnaire (TFEQ-18) was used to assess eating behaviors [32]. This is a shortened and revised version of the original 51-item TFEQ, which comprised three scales: restraint, hunger, and disinhibition [33]. We used the Polish validated version [34]. The questionnaire measures three aspects of eating behaviors: restrained eating (6 items, conscious restriction of food intake in order to control body weight or to promote weight loss), uncontrolled eating (9 items, tendency to eat more than usual due to a loss of control over intake), and emotional eating (3 items, inability to resist emotional cues) [32,35]. All items are answered on a 4-point scale (definitely true, mostly true, mostly false, definitely false), summated into scale scores for cognitive restraint, uncontrolled eating, and emotional eating and transformed to a 0–100 scale [35].

#### 2.2.4. Symptoms of Depression

The Beck Depression Inventory (BDI) [36] is the most commonly used instrument for assessing symptoms of depression. It includes twenty-one categories concerning specific behavioral manifestations of depression and consists of a graded series of self-evaluative statements. The statements are ranked to reflect the range of severity of the symptom from neutral (0) to maximal severity (3) [36]. The cut-off scores for BDI are: none or minimal depression < 10, mild to moderate depression 10–18, moderate to severe depression 19–29 and severe depression 30–63 [37].

#### 2.2.5. Diet

To assess diet, the 62-item Food Frequency Questionnaire (FFQ-6) was used. This instrument consists of a list of sixty-two food items and refers to the usual frequency of food consumption over the last 12 months [38]. Consumption of each food item is indicated by choosing one of six categories: never or very rarely, once a month or less, several times a month, several times a week, daily, or a few times a day. Subsequently, answers were converted into daily frequency. Food items were then aggregated into twenty-five food items by summing up their daily consumption frequencies (in times/day) according to Niedzwiedzka et al. [39]. Additionally, we calculated the Polish-adapted Mediterranean Diet (Polish-aMED) score according to Krusinska et al. [40]. Points were assigned for the frequency of consumption above the median for the Polish control sample for vegetables, fruit, whole grains, fish, legumes, nuts and seeds, with one extra point added for the frequency of consumption of red and processed meats below the median intake. The ‘Polish-aMED’ score was calculated as the sum of points and divided into three categories: low (0–2 points), average (3–5 points) and high (6–8 points) [40].

#### 2.2.6. Impulsiveness

The Barratt Impulsiveness Scale (BIS) is a 30-item self-report instrument designed to assess the personality/behavioral construct of impulsiveness in both research and clinical settings [41]. All items are answered on a 4-point scale (rarely/never, occasionally, often, almost always/always). The higher the summed score for all items, the higher the level of impulsiveness. Selected items were worded to indicate non-impulsiveness and scored reversely to avoid the response set [42]. The BIS has three subscales: Attentional Impulsiveness (inability to focus attention or concentrate), Motor Impulsiveness (acting without thinking), and Non-Planning Impulsiveness (lack of “futuring” or forethought) [41].

#### 2.2.7. Physical Activity

The level of physical activity was assessed using the Polish version of the International Physical Activity Questionnaire (IPAQ)—short form [43,44]. The short form IPAQ asks about three specific types of activity (walking, moderate-intensity activities, and vigorous-intensity activities) undertaken in the last 7 days. Participants were divided into three levels of physical activity: low, moderate, and high activity [44].

### 2.3. Statistical Analysis

All calculations were made using the Statistica 13 package (Statsoft, Kraków, Poland). Chi-square tests for categorical variables (being on diet, level of physical activity, adherence to Mediterranean diet, hypothyroidism), and *t*-tests for continuous variables (BMI, scores in BDI, BIS, TFEQ, FFQ) were performed. Differences of *p* < 0.05 were assumed to be statistically significant. Two categories of IPAQ (moderate and high) were merged into one category. The same procedure was employed regarding the average and high categories in the Polish-aMED. To divide participants according to the score in the Restraint Eating, Uncontrolled Eating, and Emotional Eating subscales of the TFEQ, a median split (splitting a continuous variable into high and low values) was used [45]. Participants were divided into two groups in each TFEQ subscale: Cognitive Restraint (low CR, score < 33; high CR, score ≥ 33), Uncontrolled Eating (low UE, score < 25; high UE, score ≥ 25), and Emotional Eating (low EE, score < 25; high EE, score ≥ 25).

## 3. Results

### 3.1. Participants’ Characteristics

Out of the 235 participants recruited for the study, data from eight participants were excluded from analyses due to missing survey measures (lack of information on weight or height, lack of completed BDI or TFEQ).

The final sample consisted of 227 students (44 men, 183 women). The main characteristics of the participants are summarized in Table 1.

The mean age of the participants was 21.0 (SD ± 1.2, range 19–25) and mean BMI was 22.3 kg/m^2^ (±1.2 kg/m^2^, range 15.8–38.9). Almost 16% of participants were overweight or obese. One in ten students in our sample indicated having hypothyroidism. Symptoms of at least mild depression were present in almost 35% of students (mean BDI score 7.8 ± 7.4, range 0–36), including 35.4% of physiotherapy and 34.0% of midwifery students. One-fifth of participants reported being on a diet and more than 20% smoking. More than three-quarters engaged in a moderate or high level of physical activity, according to the IPAQ score. More than 70% of participants had average to high adherence to the Mediterranean Diet. In addition, 57.2% consumed vegetables, and 44.5% ate fruits at least once a day. 52.4% ate fish at least several times per month. Only 15% of the participants consumed more vegetables, 14% fruit, and 10% of fish, in comparison to reference medians of food frequency consumption of the Polish control sample [40] (data not shown).

### 3.2. Comparison between Men and Women

The results are summarized in Table 2.

Men had significantly higher BMI than women and women had significantly higher scores in BDI. Impulsivity scores, including Non-planning Impulsivity and Attentional Impulsivity, were higher among women. As regards eating behaviors, women had significantly higher scores in emotional eating than men (26.8 ± 21.0 vs. 17.7 ± 15.5, *p* = 0.007). In terms of the FFQ scores, men consumed cheese, eggs and egg dishes, refined grain products, butter and cream, dry and processed pulses, potatoes, processed meats, red meat and venison, white meat, juices, sweetened beverages, energy drinks, and alcohol, significantly more often.

### 3.3. Comparison between Non-Overweight and Overweight/Obese Participants

Overweight/obese participants were characterized by higher scores in the emotional eating subscale of the TFEQ. They reported having hypothyroidism significantly more often. There were no significant differences in symptoms of depression, impulsivity, diet, and other eating behaviors. Data are presented in Supplementary Table S1.

### 3.4. Cognitive Restraint

Participants with high Cognitive Restraint (CR) had significantly higher BMI and a lower score in the BIS Non-planning subscale. Participants with high CR had higher adherence to the Mediterranean diet, significantly less often consumed sugar, sweets and snacks, cheese, refined grain products, butter and cream, other edible fats, potatoes, processed meats, juices, sweetened beverages, and energy drinks. Furthermore, significantly greater consumption of nuts and seeds and a tendency to eat more fruits (*p* = 0.063) in participants with high CR scores were observed. Participants with a higher cognitive restraint score were considerably more often on a diet (31.3% vs. 7.1%, *p* < 0.001) and were more physically active. Data are presented in Supplementary Table S2.

### 3.5. Uncontrolled Eating

BMI, symptoms of depression, impulsivity, including attentional impulsivity, were significantly higher in participants with high vs. low Uncontrolled Eating (UE). Participants with high UE were also notably more prone to emotional eating. In FFQ, significantly higher consumption of alcohol, sweetened milk products, and a tendency to eat more sugar, sweets, and snacks (*p* = 0.058) and eggs and egg dishes (*p* = 0.050) were observed among participants with high UE. Data are shown in Supplementary Table S3.

### 3.6. Emotional Eating

BMI, depression, and impulsiveness (tendency: total BIS score, *p* = 0.053 and significance for attentional subscale) scores were higher in participants with a high Emotional Eating (EE) score. Among eating habits, emotional eaters were also characterized by higher scores in cognitive restraint and uncontrolled eating components. Greater consumption of sugar, sweets, and snacks among participants with high emotional eating scores was observed. Data are shown in Supplementary Table S4.

## 4. Discussion

Our study was one of the first to take a multidimensional approach to the assessment of diet, physical activity, depressive symptoms, impulsivity, and eating behaviors, among students in Poland and Central Europe.

Our results revealed that more than one in three midwifery and physiotherapy students had symptoms of at least mild depression. This is in line with the results of a systematic review and meta-analysis of twenty-seven studies on the prevalence of depression in nursing students, in which a prevalence for depression of 34.0% was reported [46]. Depressive symptoms in nursing students were associated with lower academic performance [47]. Only a few studies assessed depressive symptoms in physiotherapy students. The results of a Pakistani study indicated prevalence of depressive symptoms at 48.0% [48]. Symptoms of moderate or severe depression were found in 31% of Polish physiotherapy students [49]. It is noteworthy that healthcare students with depression do not seek medical help, as depression is perceived as a stigmatizing disorder [50], which leads to self-stigma and can influence the way they will treat their future patients [51].

In our study, women had significantly higher scores in the Beck Depression Inventory than men. This is consistent with epidemiological data—women are almost twice as likely to have depression as men [52]—and also with studies carried out among students [53]. In a study by Saleh et al. [54], female university students in Paris had higher perceived stress, perceived helplessness, perceived self-efficacy, global psychological distress, somatic symptoms, anxiety, and insomnia than men.

The percentage of overweight and obese students (15.9%) was lower than indicated by the Polish statistics in the 20–29 age group (28.7%) [55], but similar to the data obtained in the group of French students [56]. Men had higher BMI than women, in line with the aforementioned Polish data [55], and studies involving university students from 22 countries [57].

Half of the participants had a moderate, and one-quarter had a high physical activity level. These finding are consistent with the results of nursing students in Scotland (76.2% students achieving 150 min of physical activity per week) [58] and to those of biomedical students in Australia (more than 80% of students achieving sufficient levels of physical activity). The activity of our students was higher than that of health science students in Lebanon (48.2% with a moderate and 10.6% with a high activity level) [59], and of medical students in Sudan (32.0% with a moderate and 23.1% with a high physical activity level) [60]. In our study, the level of physical activity did not vary significantly between males and females, as in a study by Chourdakis et al. [61], although other research points to higher physical activity among men [60,62,63,64].

In our study, almost three-quarters of students had average to high adherence to the Mediterranean diet, which has been shown to reduce the burden or even prevent the development of cardiovascular disease, several cancers, depression, diabetes, obesity, and cognitive decline [65]. However, only approximately half of the participants in our study consumed at least one serving of vegetables and fruit daily. In a study by Gallo et al. [66], among Australian students, only 1 in 10 students met fruit or vegetable recommendations, with less than one-third met recommendations for fiber, calcium, and potassium. According to the joint WHO/FAO Expert Consultation Report [67], a daily intake of fresh fruit and vegetables in an adequate quantity (400–500 g) is recommended to reduce the risk of coronary heart disease, stroke, and high blood pressure. According to Polish dietary guidelines, 400 g of vegetables and fruit a day should be eaten [68]. In a longitudinal study, carried out in a large cohort, a higher intake of fruit and vegetables was associated with lower mortality; the thresholds of risk reduction in mortality were two servings for fruit intake daily and three servings for vegetable intake daily [69]. In our study, only half of the participants ate fish at least several times a month. Similarly, Muñoz-Rodríguez et al. [70] reported unsatisfactory consumption levels of fruit, vegetables, and fish in Spanish university students. Regular fish consumption (1–2 servings per week) protects against coronary heart disease and ischemic stroke and is, thus, recommended by the WHO [67] and Polish guidelines [68]. However, there is a growing discussion on the consumption of fish and other seafood due to the pressure on natural resources and challenges for the sustainability of marine and inland fisheries [71,72]; therefore, further research is needed on possible replacements for fish in healthy, sustainable diets.

Our study showed that men significantly more often than women consumed high-fat products, meat, sweetened beverages, energy drinks, and alcohol. This is consistent with other studies, reporting that males’ diet is of inferior quality; they consume more meat, high-fat products and have less healthy eating habits [64,73,74], and the results of the review of Wicki et al., among students at European universities [75], indicate greater consumption of alcohol in men.

We also confirmed the observation by Wardle et al., that female students more often than men reported avoiding high-fat foods, eating fruit and fiber, and attached greater importance to healthy eating [57]. In our study, the rates of dieters and non-dieters did not differ between males and females, as opposed to the study by Wardle [57], in which women were more likely to be dieting. We also did not confirm the results of studies indicating that women are more likely to consume sweets [74,76,77].

Our results concerning higher emotional eating in women are consistent with other studies [35,56,78,79,80]. However, we did not observe higher cognitive restraint in the female group, reported in previous studies [35,81].

In our sample, participants with higher cognitive restraint had higher adherence to the Mediterranean diet than the remaining students. Their diet was of overall better quality, contained less sugar, fat, and processed meat; they less often drank juices, sweetened beverages, and energy drinks. Additionally, they consumed nuts and seeds more often, tended to eat more fruit, and were more physically active. In a study by Abdella et al. [76], increased cognitive restraint was correlated with lower cravings for fatty foods, sweet foods, and fast foods; similarly, in a study by de Lauzon et al., high restrainers reported to preferentially consume healthy food, such as fish, vegetables, and fat-reduced food [35].

However, cognitive restraint was connected with higher BMI, which is consistent with some previous research [79,80,82,83,84]; opposite results were also reported [85]. Association between CR and obesity is complex. In a study by van Strien et al., adult women with higher dietary restraint scores had an increased risk of developing obesity [86]. It is also possible that people with a higher body mass index are more concerned with their dietary intake and are actively trying to control or reduce their weight and restraint eating is a way to control food cravings and diet effectively [76,82]. In a study by de Lauzon-Guillain et al. [87], which assessed the relations between eating behavior and adiposity in a general population over a 2-year period, a higher initial BMI was associated with a greater increase in the cognitive restraint score in all subjects. Higher restraint eating did not lead to an increase in adiposity over time. Similarly, in a longitudinal study among adolescents by Snoek et al. [88], restraint eating was neither a predictor of weight increase nor an effective weight loss strategy, suggesting that a higher BMI predicted more restraint eating.

Uncontrolled eating was associated with a higher BMI, higher consumption of alcohol, and a tendency to eat more sugar, sweets, and snacks. Higher BMI among uncontrolled eaters was demonstrated in previous studies [82,83,89] and our results add to these. In a study by de Lauzon et al. [35], in the teenagers and young adult group, higher uncontrolled eaters reported a higher alcoholic beverage intake than medium uncontrolled eaters, whereas Aoun et al. observed this relationship only in males [79]. Moreover, increased uncontrolled eating was associated with increased cravings for carbohydrates in a study by Abdella et al. [76].

In our study, the emotional eating score was strongly associated with depressive symptoms, which is in line with data from other authors, showing a relationship between EE and negative emotions and perceived stress [56,90,91]. In our sample, emotional eaters more often consumed sugar, sweets and snacks, consistent with previous reports [43,92]. Moreover, we confirmed previous results on the associations of emotional eating with higher BMI [56,79,80,82,83,89,90].

In our study, higher impulsivity was characteristic of women and people with high emotional and uncontrolled eating levels. On the other hand, a higher score in cognitive restraint was associated with a lower score on the Non-planning subscale of the Barratt Impulsivity Scale. Sex differences in impulsivity are not fully elucidated (previous studies indicated higher impulsivity among women [93], men [94] or did not reveal significant differences [85,95]). Attentional Impulsiveness (inability to focus attention or concentrate) was linked to emotional eating in previous studies [85,96]. In a study by Jasinska et al., Attentional and Motor Impulsiveness were associated with external eating (overeating in response to external food cues, measured by the Dutch Eating Behavior Questionnaire) [85]. In a systematic review by Maxwell et al., self-reported impulsivity was associated with food addiction [92], and Flaudias et al. [97] indicated a strong association between impulsivity and alcohol consumption among students.

Our research is not without its limitations—the cross-sectional design and a relatively small number of participants involved. Another limitation is that the sample is imbalanced in terms of male and female ratio, due to the higher number of females studying physiotherapy and midwifery. Additionally, our findings are based on a sample of university students in their twenties, and generalization to other population groups should be made with caution. This study focused only on physiotherapy and midwifery students and employed a non-random sampling method, which further limits the generalizability of the results. Finally, we used self-reported data on weight and height of participants (participants’ BMI may be underestimated).

## 5. Strengths of the Study and Future Directions

An important strength of our study is the multidimensional and comprehensive assessment of eating behaviors, depressive symptoms, physical activity, and diet in healthcare students. Previous studies focused mainly on single symptoms or health behaviors as separate entities. It is noteworthy that most adults report habitual participation in more than one health-risk behavior [98]. Therefore, it is not enough to focus only on physical activity or a selected aspect of dietary behavior; co-occurrence of unhealthy behaviors should be studied. Addressing multiple health behaviors has a greater impact on health and well-being than changing a single behavior alone [99].

The second strength of our study is its focus on the psychological functioning of healthcare students. The high prevalence of depressive symptoms indicates that universities should assist students in seeking professional help, through student mental health centers and universal mental health screening programs. Additionally, depressive symptoms may negatively influence students’ health behaviors (diet, physical activity). In our study, an association between emotional and uncontrolled eating and depressive symptoms was found. Both emotional and uncontrolled eating can lead to overeating and obesity; thus, psychological aspects and eating behaviors should be considered in the development of lifestyle interventions.

Thirdly, our study brings attention to the lifestyle of future health professionals. Physiotherapy and midwifery students will be role models and promote a healthy life-style, including healthy diet and physical activity. Previous reports suggest that active healthcare professionals are more likely to provide better and more credible preventive counseling; in contrast, physicians having unhealthy habits tend to have less effective dialogues with patients to motivate them [100,101]. To be credible role models for the patients, healthcare professionals need to have a healthy lifestyle themselves [102].

Universities are an ideal setting for the implementation of health promotion pro-grams, as they are a learning environment, supporting a large student population, at a key time for the development of lifestyle skills and behaviors [103]. Further research is needed due to the heterogeneity of the previous findings. This study can be a starting point to further research on the mental health and lifestyle of emerging adults, in order to develop tailored interventions aimed at students’ mental wellbeing and the implementation of healthy lifestyle modifications. Multicenter observational follow-up programs, assessing multiple lifestyle factors (dietary intake, physical activity, sedentary behavior, smoking and drinking habits, sleeping patterns) and mental health are necessary.

## 6. Conclusions

Our results indicate the need for interventions, targeting mental health, nutrition, and obesity prevention in the university setting. It is worth noting that, in our sample, one in three students exhibited depressive symptoms, implying a need to strengthen early screening toward depression and other mental problems and provide access to mental health services in university settings. We also note that one in ten students in the sample had hypothyroidism, and an association between hypothyroidism and overweight/obesity was also observed. One in four participants showed a low level of physical activity. More than 40% of the students did not consume vegetables at least once a day, and more than half did not consume fruit once daily. Only approximately 50% of the students ate fish several times a month. An association was observed between high scores of specific eating behaviors and body weight, adherence to the Mediterranean diet, and consumption of specific product groups (sweets, alcohol). We hope that our work will contribute to increasing the body of knowledge on the determinants of overweight and obesity in young adults and provide a starting point for further research in this field. Our research indicates the complex nature of students’ eating behaviors and the role of psychological factors in their emergence, so interventions cannot simply provide information on recommended physical activity and appropriate diet. Both prevention and treatment of obesity require the involvement and collaboration of a multidisciplinary team, comprising specialists in medicine, dietetics, psychology, and public health. It is worth emphasizing that interventions in the university setting can impact the whole community of university students. This is particularly important in the case of medical schools, where students, as in other universities, are future employees and parents, but are also future health care professionals, who will be dealing with, among others, obesity issues. Therefore, their knowledge and attitudes towards overweight and obese people will be essential in their professional work.

## Figures and Tables

**Table 1:** Characteristics of the participants.

Characteristic	*n*	%
Study course		
Physiotherapy	130	57.3
Midwifery	97	42.7
Sex		
Men	44	19.4
Women	183	80.6
Weight status (BMI)		
Underweight	22	9.7
Normal	169	74.5
Overweight	25	11.0
Obese	11	4.9
Hypothyroidism		
Yes	21	9.3
No	206	90.8
On diet		
Yes	47	20.7
No	180	79.3
Smoking		
Yes	48	21.1
No	179	78.9
Symptoms of depression (BDI)		
No/minimal	148	65.2
Mild	57	25.1
Moderate	16	7.0
Severe	6	2.6
Level of physical activity (IPAQ)		
Low	51	22.5
Moderate	118	52.0
High	58	25.6
Level of adherence to Mediterranean Diet (Polish-aMED)		
Low	61	26.9
Average	136	59.9
High	30	13.2

BMI—Body Mass Index; BDI—Beck Depression Inventory; IPAQ—International Physical Activity Questionnaire; Polish-aMED—Polish-adapted Mediterranean Diet.

**Table 2:** Participants’ body mass index, symptoms of depression, impulsiveness, eating behaviors, diet, hypothyroidism, dieting and physical activity broken down by gender; males *n* = 44, females *n* = 183.

	Males		Females		
	Mean	SD	Mean	SD	*p*
Body Mass Index	23.4	3.4	22.1	3.7	0.035
Beck Depression Inventory	5.3	6.4	8.4	7.5	0.010
Barratt Impulsiveness Scale					
Total	56.6	9.4	60.9 ^b^	9.5 ^b^	0.007
Non-planning	21.7	4.5	23.4 ^b^	4.4 ^b^	0.017
Motor	18.6	3.5	19.4 ^b^	4.2 ^b^	n.s.
Attentional	16.6	4.1	18.5 ^b^	3.7 ^b^	0.003
Three-Factor Eating Questionnaire					
Cognitive Restraint	29.9	13.7	34.1	14.5	n.s.
Uncontrolled Eating	24.9	12.7	24.3	13.0	n.s.
Emotional Eating	17.7	15.5	26.8	21.0	0.007
Food Frequency Questionnaire (aggregated)					
Sugar, sweets and snacks	1.7	1.3	1.6	1.3	n.s.
Milk, fermented milk drinks and curd cheese	1.3	0.8	1.2	0.8	n.s.
Sweetened milk products	0.4	0.5	0.4	0.5	n.s.
Cheese	0.8	0.5	0.6	0.5	0.022
Eggs and egg dishes	0.6	0.5	0.4	0.4	0.041
Breakfast cereals	0.2	0.3	0.2	0.2	n.s.
Whole grain products	0.8	0.6	0.9	0.6	n.s.
Refined grain products	1.0	0.6	0.8 ^c^	0.6 ^c^	0.008
Butter and cream	0.9	0.6	0.7 ^b^	0.7 ^b^	0.035
Other animal fats	0.0	0.1	0.0	0.1	n.s.
Vegetable oils	0.6	0.5	0.5	0.4	n.s.
Other edible fats	0.4	0.5	0.3	0.4	n.s.
Fruits	0.8	0.6	0.8 ^c^	0.5 ^c^	n.s.
Dried fruit, fruit preserves and fruit condiments	0.3	0.4	0.3	0.4	n.s.
Vegetables	0.9	0.5	0.9	0.5	n.s.
Dry and processed pulses	0.3	0.4	0.1	0.2	0.005
Potatoes	0.5	0.4	0.4	0.3	0.005
Nuts and seeds	0.5	0.5	0.4	0.5	n.s.
Processed meats	1.0	0.9	0.6 ^b^	0.6 ^b^	0.000
Red meat and venison	0.4	0.4	0.1 ^b^	0.2 ^b^	0.000
White meat	0.5	0.3	0.3 ^b^	0.3 ^b^	0.025
Fish	0.2	0.3	0.2 ^b^	0.3 ^b^	n.s.
Juices	0.6	0.6	0.4 ^b^	0.5 ^b^	0.017
Sweetened beverages and energy drinks	0.3	0.6	0.1 ^a^	0.3 ^a^	0.002
Alcohol	0.4	0.6	0.3 ^a^	0.3 ^a^	0.009
	***n***	**%**	***n***	**%**	***p***
Hypothyroidism					
Yes	3	6.8	18	9.8	n.s.^Y^
No	41	93.2	165	90.2	
On diet					
Yes	10	22.7	37	20.2	n.s.
No	34	77.3	146	79.8	
International Physical Activity Questionnaire					
Low activity	8	18.2	43	23.5	n.s.
Moderate-high activity	36	81.8	140	76.5	
Polish-adapted Mediterranean Diet					
Low adherence	12	27.3	49	26.8	n.s.
Average-high adherence	32	72.7	134	73.2	

SD—standard deviation; ^a^ *n* = 180; ^b^ *n* = 181; ^c^ *n* = 182; ^Y^ Yates’ correction.

## Data Availability

The data presented in this study are available upon request from the corresponding author.

## Supplementary Materials

The following are available online at https://www.mdpi.com/article/10.3390/nu14051106/s1, Table S1. Participants’ body mass index, symptoms of depression, impulsiveness, eating behaviors, diet, hypothyroidism, dieting and physical activity broken down by Body Mass Index, Table S2. Participants’ body mass index, symptoms of depression, impulsiveness, eating behaviors, diet, hypothyroidism, dieting and physical activity broken down by Cognitive Restraint (CR)—median split, Table S3. Participants’ body mass index, symptoms of depression, impulsiveness, eating behaviors, diet, hypothyroidism, dieting and physical activity broken down by Uncontrolled Eating (UE)—median split, Table S4. Participants’ body mass index, symptoms of depression, impulsiveness, eating behaviors, diet, hypothyroidism, dieting and physical activity broken down by Emotional eating (EE)—median split.
