# Supplementary material for: Eating Behaviors, Depressive Symptoms and Lifestyle in University Students in Poland
Source: Nutrients. 2022 Mar 6;14(5):1106. doi: 10.3390/nu14051106 (PMC8912316; doi:10.3390/nu14051106)
Supplement: Supplementary file 1 [file nutrients-14-01106-s001.zip › nutrients-1575912-supplementary.pdf]

nutrients

Supplementary

Eating Behaviors and Lifestyle in Polish Students

Julia Suwalska [1,*], Kalina Kolasińska [2], Dorota Łojko [3] and Paweł Bogdański [1]

Table S1. Participants' body mass index, symptoms of depression, impulsiveness, eating behaviors, diet, hypothyroidism, dieting and physical activity broken down by Body Mass Index; non-overweight $n = 191$, overweight/obese $n = 36$.

	Non-overweight		Overweight/obese		
	**Mean**	**SD**	**Mean**	**SD**	***p***
Body Mass Index	21.1	2.1	28.8	3.6	0.000
Beck Depression Inventory	7.8	7.3	7.9	7.8	n.s.
Barratt Impulsiveness Scale					
Total	23.2[a]	4.5[a]	22.4	3.9	n.s.
Non-planning	19.3[a]	4.2[a]	19.2	3.7	n.s.
Motor	18.0[a]	3.8[a]	18.5	4.1	n.s.
Attentional	60.1[a]	9.7[a]	59.6	9.1	n.s.
Three-Factor Eating Questionnaire					
Cognitive Restraint	33.0	14.6	34.7	13.7	n.s.
Uncontrolled Eating	24.0	12.7	26.6	14.0	n.s.
Emotional Eating	23.1	19.5	35.2	21.8	0.001
Food Frequency Questionnaire (aggregated)					
Sugar, sweets and snacks	1.7	1.3	1.5	1.2	n.s.
Milk, fermented milk drinks and curd cheese	1.2	0.8	1.4	0.9	n.s.
Sweetened milk products	0.4	0.5	0.4	0.4	n.s.
Cheese	0.6	0.5	0.6	0.5	n.s.
Eggs and egg dishes	0.4	0.4	0.4	0.4	n.s.
Breakfast cereals	0.2	0.3	0.2	0.3	n.s.
Whole grain products	0.9	0.7	0.9	0.6	n.s.
Refined grain products	0.9[b]	0.6[b]	0.7	0.5	n.s.
Butter and cream	0.8[a]	0.7[a]	0.7	0.5	n.s.
Other animal fats	0.0	0.1	0.0	0.1	n.s.
Vegetable oils	0.5	0.4	0.4	0.4	n.s.
Other edible fats	0.3	0.4	0.2	0.3	n.s.
Fruits	0.8[b]	0.5[b]	0.9	0.6	n.s.
Dried fruit, fruit preserves and fruit condiments	0.3	0.4	0.2	0.3	n.s.
Vegetables	0.9	0.5	0.8	0.6	n.s.
Dry and processed pulses	0.2	0.3	0.2	0.2	n.s.
Potatoes	0.4	0.3	0.3	0.3	n.s.
Nuts and seeds	0.5	0.5	0.4	0.4	n.s.
Processed meats	0.7[b]	0.7[b]	0.7[c]	0.6[c]	n.s.
Red meat and venison	0.2[b]	0.3[b]	0.2[c]	0.2[c]	n.s.
White meat	0.4[b]	0.3[b]	0.3[c]	0.2[c]	n.s.
Fish	0.2[b]	0.3[b]	0.1[c]	0.2[c]	n.s.
Juices	0.4[b]	0.5[b]	0.4[c]	0.5[c]	n.s.
Sweetened beverages and energy drinks	0.2[a]	0.4[a]	0.2[c]	0.3[c]	n.s.
Alcohol	0.3[a]	0.4[a]	0.4[c]	0.4[c]	n.s.
	n	%	*n*	%	*p*

Hypothyroidism					
Yes	14	7.3	7	19.4	0.021
No	177	92.7	29	80.6	
On diet					
Yes	39	20.4	8	22.2	n.s.
No	152	79.6	28	77.8	
International Physical Activity Questionnaire					
Low activity	41	21.5	10	27.8	n.s.
Moderate-high activity	150	78.5	26	72.2	
Polish-adapted Mediterranean Diet					
Low adherence	53	27.7	8	22.2	n.s.
Average-high adherence	138	72.3	28	77.8	

SD – standard deviation; [a] n = 189; [b] n = 190; [c] n = 35.

Table S2. Participants' body mass index, symptoms of depression, impulsiveness, eating behaviors, diet, hypothyroidism, dieting and physical activity broken down by Cognitive Restraint (CR) – median split; low CR *n* = 99, high CR *n* = 128.

	**Low CR**		**High CR**		
	**Mean**	**SD**	**Mean**	**SD**	***p***
Body Mass Index	21.7	3.6	22.8	3.7	0.025
Beck Depression Inventory	6.9	7.1	8.6	7.5	n.s.
Barratt Impulsiveness Scale					
Total	60.7[a]	9.9	59.6	9.3	n.s.
Non-planning	23.8[a]	4.6	22.5	4.2	0.022
Motor	19.4[a]	4.4	19.2	3.9	n.s.
Attentional	17.6[a]	3.8	18.5	3.8	n.s.
Three-Factor Eating Questionnaire					
Cognitive Restraint	20.2	8.1	43.4	9.1	0.000
Uncontrolled Eating	24.0	14.2	24.8	11.9	n.s.
Emotional Eating	21.3	21.2	27.9	19.2	0.015
Food Frequency Questionnaire (aggregated)					
Sugar, sweets and snacks	1.9	1.4	1.4	1.1	0.002
Milk, fermented milk drinks and curd cheese	1.2	0.7	1.3	0.8	n.s.
Sweetened milk products	0.4	0.5	0.3	0.5	n.s.
Cheese	0.7	0.5	0.5	0.4	0.003
Eggs and egg dishes	0.4	0.5	0.4	0.4	n.s.
Breakfast cereals	0.2	0.2	0.2	0.3	n.s.
Whole grain products	0.8	0.7	0.9	0.6	n.s.
Refined grain products	1.0[b]	0.6	0.7	0.5	0.000
Butter and cream	1.0	0.8	0.6[c]	0.6	0.000
Other animal fats	0.0	0.1	0.0	0.1	n.s.
Vegetable oils	0.6	0.5	0.5	0.4	n.s.
Other edible fats	0.4	0.5	0.2	0.3	0.000
Fruits	0.8	0.5	0.9[d]	0.6	n.s.
Dried fruit, fruit preserves and fruit condiments	0.3	0.4	0.3	0.4	n.s.
Vegetables	0.9	0.5	0.9	0.5	n.s.
Dry and processed pulses	0.1	0.2	0.2	0.3	n.s.
Potatoes	0.4	0.3	0.3	0.3	0.023
Nuts and seeds	0.3	0.4	0.5	0.6	0.008
Processed meats	0.8[b]	0.8	0.5[d]	0.6	0.002
Red meat and venison	0.2[b]	0.3	0.2[d]	0.2	n.s.
White meat	0.4[b]	0.3	0.4[d]	0.3	n.s.
Fish	0.1[b]	0.2	0.2[d]	0.3	n.s.
Juices	0.5[b]	0.6	0.3[c]	0.5	0.043
Sweetened beverages and energy drinks	0.3[b]	0.5	0.1[c]	0.3	0.012
Alcohol	0.3[b]	0.3	0.3[c]	0.4	n.s.
	n	%	*n*	%	*p*
Hypothyroidism					
Yes	7	7.1	14	10.9	n.s.
No	92	92.9	114	89.1	
On diet					
Yes	7	7.1	40	31.3	0.000
No	92	92.9	88	68.8	
International Physical Activity Questionnaire					
Low activity	29	29.3	22	17.2	0.030
Moderate-high activity	70	70.7	106	82.8	
Polish-adapted Mediterranean Diet					

Low adherence	35	35.4	26	20.3	0.001
Average-high adherence	64	64.6	128	79.7	

SD – standard deviation; [a] *n* = 97; [b] *n* = 98; [c] *n* = 126; [d] *n* = 127;
Cognitive Restraint: low CR – score <33, high CR – score ≥33.

Table S3. Participants' body mass index, symptoms of depression, impulsiveness, eating behaviors, diet, hypothyroidism, dieting and physical activity broken down by Uncontrolled Eating (UE) – median split; low UE *n* = 111, high UE *n* = 116.

	Low UE		High UE		
	**Mean**	**SD**	**Mean**	**SD**	***p***
Body Mass Index	21.6	3.2	23.1	4.0	0.002
Beck Depression Inventory	6.8	7.1	8.8	7.6	0.037
Barratt Impulsiveness Scale					
Total	58.2[a]	10.1	61.8	8.8	0.005
Non-planning	22.6[a]	4.5	23.5	4.3	n.s.
Motor	19.0[a]	4.4	19.5	3.9	n.s.
Attentional	16.8[a]	3.6	19.4	3.6	0.000
Three-Factor Eating Questionnaire					
Cognitive Restraint	32.2	14.9	34.3	13.9	n.s.
Uncontrolled Eating	14.2	6.5	34.3	9.5	0.000
Emotional Eating	15.0	14.4	34.6	20.6	0.000
Food Frequency Questionnaire (aggregated)					
Sugar, sweets and snacks	1.5	1.2	1.8	1.3	n.s.
Milk, fermented milk drinks and curd cheese	1.2	0.8	1.2	0.8	n.s.
Sweetened milk products	0.3	0.4	0.4	0.5	0.021
Cheese	0.6	0.5	0.6	0.4	n.s.
Eggs and egg dishes	0.4	0.4	0.5	0.5	n.s.
Breakfast cereals	0.2	0.3	0.2	0.2	n.s.
Whole grain products	0.9	0.7	0.9	0.6	n.s.
Refined grain products	0.8[b]	0.6	0.9	0.5	n.s.
Butter and cream	0.8[b]	0.7	0.7[e]	0.7	n.s.
Other animal fats	0.0	0.1	0.0	0.1	n.s.
Vegetable oils	0.5	0.4	0.6	0.4	n.s.
Other edible fats	0.3	0.5	0.3	0.4	n.s.
Fruits	0.8	0.5	0.9[e]	0.6	n.s.
Dried fruit, fruit preserves and fruit condiments	0.2	0.4	0.3	0.5	n.s.
Vegetables	1.0	0.6	0.9	0.5	n.s.
Dry and processed pulses	0.2	0.3	0.2	0.3	n.s.
Potatoes	0.4	0.3	0.4	0.3	n.s.
Nuts and seeds	0.4	0.4	0.5	0.5	n.s.
Processed meats	0.7	0.7	0.7	0.7	n.s.
Red meat and venison	0.2	0.2	0.2[d]	0.3	n.s.
White meat	0.4	0.3	0.4[d]	0.3	n.s.
Fish	0.2	0.3	0.2[d]	0.3	n.s.
Juices	0.4	0.5	0.4[d]	0.5	n.s.
Sweetened beverages and energy drinks	0.2	0.4	0.2[c]	0.3	n.s.
Alcohol	0.2	0.3	0.4[c]	0.4	0.004
	n	%	*n*	%	*p*
Hypothyroidism					
Yes	14	12.6	7	6.0	n.s.
No	97	87.4	109	94.0	
On diet					
Yes	23	20.7	24	20.7	n.s.
No	88	79.3	92	79.3	
International Physical Activity Questionnaire					
Low activity	27	24.3	24	20.7	n.s.
Moderate-high activity	84	75.7	92	79.3	
Polish-adapted Mediterranean Diet					

Low adherence	30	27.0	31	26.7	n.s.
Average-high adherence	81	73.0	85	73.3	

SD – standard deviation; [a] *n* = 109; [b] *n* = 110; [c] *n* = 113; [d] *n* = 114; [e] *n* =115;
Uncontrolled Eating: low UE – score <25, high UE – score ≥25.

Table S4. Participants' body mass index, symptoms of depression, impulsiveness, eating behaviors, diet, hypothyroidism, dieting and physical activity broken down by Emotional eating (EE) – median split; low EE *n* = 103, high EE *n* = 124.

	Low EE		High EE		
	**Mean**	**SD**	**Mean**	**SD**	***p***
Body Mass Index	21.1	2.8	23.4	4.0	0.000
Beck Depression Inventory	6.2	6.7	9.2	7.7	0.002
Barratt Impulsiveness Scale					
Total	58.7[a]	11.0	61.2	8.2	n.s.
Non-planning	22.7[a]	4.7	23.4	4.2	n.s.
Motor	19.1[a]	4.8	19.4	3.5	n.s.
Attentional	17.1[a]	3.8	18.9	3.7	0.000
Three-Factor Eating Questionnaire					
Cognitive Restraint	30.9	15.5	35.3	13.3	0.023
Uncontrolled Eating	18.0	10.9	29.8	12.1	0.000
Emotional Eating	7.6	7.7	39.5	15.6	0.000
Food Frequency Questionnaire (aggregated)					
Sugar, sweets and snacks	1.4	1.2	1.9	1.3	0.002
Milk, fermented milk drinks and curd cheese	1.2	0.8	1.3	0.8	n.s.
Sweetened milk products	0.3	0.5	0.4	0.5	n.s.
Cheese	0.6	0.5	0.6	0.5	n.s.
Eggs and egg dishes	0.4	0.4	0.4	0.4	n.s.
Breakfast cereals	0.2	0.3	0.2	0.2	n.s.
Whole grain products	0.9	0.7	0.9	0.6	n.s.
Refined grain products	0.9[b]	0.6	0.8	0.5	n.s.
Butter and cream	0.8	0.7	0.7[d]	0.6	n.s.
Other animal fats	0.0	0.1	0.0	0.1	n.s.
Vegetable oils	0.5	0.4	0.5	0.4	n.s.
Other edible fats	0.3	0.5	0.3	0.3	n.s.
Fruits	0.8	0.5	0.9[e]	0.6	n.s.
Dried fruit, fruit preserves and fruit condiments	0.3	0.4	0.3	0.4	n.s.
Vegetables	1.0	0.5	0.9	0.5	n.s.
Dry and processed pulses	0.2	0.3	0.2	0.2	n.s.
Potatoes	0.4	0.4	0.4	0.3	n.s.
Nuts and seeds	0.4	0.4	0.5	0.5	n.s.
Processed meats	0.7	0.8	0.6[d]	0.6	n.s.
Red meat and venison	0.2	0.3	0.2[d]	0.3	n.s.
White meat	0.3	0.3	0.4[d]	0.3	n.s.
Fish	0.2	0.2	0.2[d]	0.3	n.s.
Juices	0.4	0.6	0.4[d]	0.5	n.s.
Sweetened beverages and energy drinks	0.2	0.4	0.2[c]	0.3	n.s.
Alcohol	0.3[b]	0.4	0.3[d]	0.4	n.s.
	n	%	*n*	%	*p*
Hypothyroidism					
Yes	9	8.7	12	9.7	n.s.
No	94	91.3	112	90.3	
On diet					
Yes	24	23.3	23	18.5	n.s.
No	79	76.7	101	81.5	
International Physical Activity Questionnaire					
Low activity	23	22.3	28	22.6	n.s.
Moderate-high activity	80	77.7	96	77.4	
Polish-adapted Mediterranean Diet					

Low adherence	27	26.2	34	27.4	n.s.
Average-high adherence	76	73.3	90	72.6	

SD – standard deviation; [a] n = 101; [b] n = 102; [c] n = 121; [d] n = 122; [e] n = 123;
Emotional Eating: low EE – score <25, high EE – score ≥25.
